# Influences of Chemical Composition and Fineness on the Development of Concrete Strength by Curing Conditions

**DOI:** 10.3390/ma12244061

**Published:** 2019-12-05

**Authors:** Jaehyun Lee, Taegyu Lee

**Affiliations:** Technology Research and Development Institute, Daelim Industrial, Jongno-Gu, Seoul 03152, Korea; archi0528@daum.net

**Keywords:** chemical composition, fineness, setting time, early compressive strength, high SO_3_ Portland cement (HSPC), ordinary Portland cement (OPC), form removal time

## Abstract

In this study, the influences of chemical composition and fineness on the development of concrete strength by curing conditions were investigated through performance evaluation of high SO_3_ Portland cement (HSPC) and ordinary Portland cement (OPC). At the same fineness (3800 cm^2^/g), the initial and final setting times of HSPC were 92 and 98 min less than OPC. Early mortar compressive strength was approximately 176% higher after 24 h. After curing for 15 h, 18 h, and 24 h, the maturity of HSPC concrete (107.4%, 109.6%, and 111.7%) and early compressive strength (146.4%, 170.7%, and 154.5%) were higher than measured for OPC concrete. HSPC fineness was 111.8% higher than OPC, leading to early activation of the hydration reaction. By X-ray fluorescence analysis, the SO_3_ content of HSPC was 107.9% that of OPC. The applicable time for HSPC concrete form removal was shorter than that for OPC concrete. The relationships y = −10.57 ln(x) + 47.30 and y = −9.84 ln(x) + 44.05 were estimated for predicting the early-age strength OPC and HSPC concrete. Therefore, applying HSPC concrete to an actual construction site is expected to shorten the construction period and reduce the heating curing cost in winter compared to OPC concrete.

## 1. Introduction

Concrete is the most commonly used construction material because of its excellent strength and durability [1,2]. After a concrete structure has cured, the removal of the forms used to hold its shape during hardening is the most important part of the management process at construction sites that must adhere to the standards determined during the audit process. Therefore, predicting the early-age strength of concrete by calculating the relationship between the curing temperature and mechanical strength is becoming an increasingly important part of concrete research [3,4]. Several recent publications have employed the maturity method to estimate the compressive strength of different types of concrete, including mass concrete [5,6], steel fiber reinforced concrete [7], sprayed concrete [8], and eco-concrete [9,10,11]. However, to the best of our knowledge, no previous studies have estimated the compressive strength of field applicable high SO_3_ Portland cement (HSPC).

The contribution of cement particles to the strength of the concrete is closely related to their size [12,13]. Specifically, cement particle size controls the degree of hydration in the early hydration stage; i.e., finer cement particles lead to higher heat of hydration [14,15], with extremely fine cement particles being completely hydrated within 24 h [16]. Conversely, finer cement grains can affect the generation of early-age cracks in the cement matrix [17,18]. This study evaluated the performance of cement with a higher degree of fineness than ordinary Portland cement (OPC).

Superplasticizers are also applied to cementitious materials and concrete fabrication to realize significant improvements in machinability, mechanical properties, porosity, and durability. They can provide excellent workability while substantially reducing the water/binder ratio [19,20,21,22] and increasing strength. This takes place up to a point beyond which low water/binder ratios can lead to other problems. For example, the degree of hydration of cement minerals may be significantly reduced. Consequently, their ability to directly contribute to cement performance will also be reduced; therefore, the plasticizer addition level must be optimized [23].

Portland cement CEM I is a powdered material made of clinker and gypsum [24]. Gypsum is used to control the hardening time of cement [25], and the mechanical properties (strength, shrinkage, and expansion) of cement can change depending on the amount of gypsum in the matrix [26,27]. Gypsum may be present in small proportions as anhydrite (CaSO_4_) but, owing to instability, rarely as the hemihydrate (CaSO_4_∙1/2H_2_O). Gypsum is present in cement as calcium sulfate dihydrate (CaSO_4_∙2H_2_O) and is expressed in terms of trioxide sulfate (SO_3_) levels [28]. The SO_3_ content should be less than 4% according to European standard EN 197-1 [29] because, in cementitious systems, low SO_3_ content cannot guarantee sufficient condensation delay, whereas high SO_3_ content has a significant effect on the cement strength and dimensional stability. Optimal SO_3_ content ensures maximum strength and minimum shrinkage of the matrix without excessive expansion in water [26,30,31].

According to Kurdowski [32], the optimum SO_3_ content (%) can be calculated using one of the following formulas:

% SO_3(Optimum)_ = 0.556∙Na_2_O_2_ + 0.0017659∙Fineness + 0.1072∙Fe_2_O_3_ − 3.6004 (1)

% SO_3(Optimum)_ = 0.093∙Al_2_O_3_ + 1.71∙Na_2_O_2_ + 0.94∙K_2_O + 1.23 (2)

% SO_3(Optimum)_ = 6.810^−5^∙Fineness∙C_3_A (3)

% SO_3(Optimum)_ = 1.841 + 0.095∙C_3_A + 1.6364∙Na_2_O_2_. (4)

**Figure 1:**
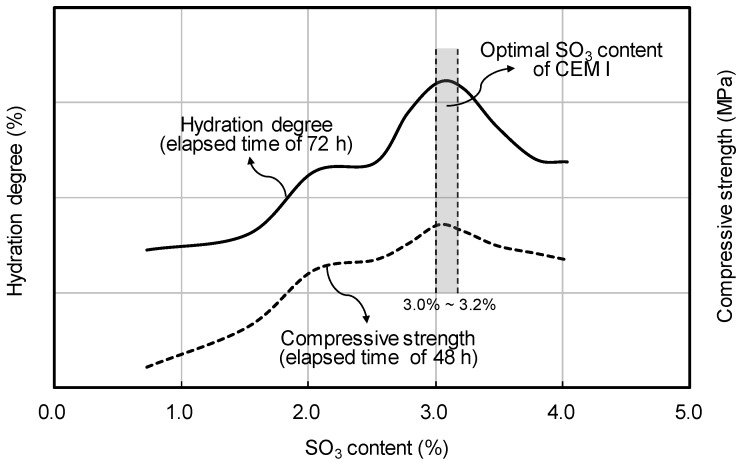
Preliminary test results of the compressive strength of fine cement according to binder weight and curing temperature.

However, as reported by Mohammed and Safiullah [33], and shown in Figure 1, it has been proven through testing that the maximum hydration degree (elapsed time of 72 h) and compressive strength (elapsed time of 48 h) are increased when SO_3_ content is approximately 3.0%–3.2%.

To maximize early compressive strength and apply the findings to construction sites, the research team added gypsum to OPC clinker, increasing SO_3_ to 3.1% and improving fineness by 111.8% (400 cm^2^/g on 3400 cm^2^/g). In this study, the new HSPC was evaluated against OPC by setting time and early compressive strength according to cement fineness and type in a mortar test (Series I), and slump and air content according to cement fineness and type in a concrete test (Series II). Early compressive strength and maturity were evaluated in the Series II testing. The strength prediction of concrete and the possibility of removing the vertical formwork according to the heat of hydration were applied to the in-place curing of concrete samples in a field test structure.

## 2. Experimental Procedure

### 2.1. Materials

Ordinary type 1 Portland cement and type 2 fly ash (FA) that met the KS L 5201 standard (Portland cement) [34] were used as binders (Table 1). HSPC cement with fineness increased to approximately 111.8% (400 cm^2^/g) was prepared by adding gypsum to a level of 3.1% SO_3_ content to ensure the development of early compressive strength. Washed sea sand (40%) and crushed sand (60%) were mixed for use as the fine aggregate. Crushed granite aggregate was used as the coarse aggregate. Polycarboxylic superplasticizer was applied to both OPC and HSPC, as the only admixture used.

**Figure 2:**
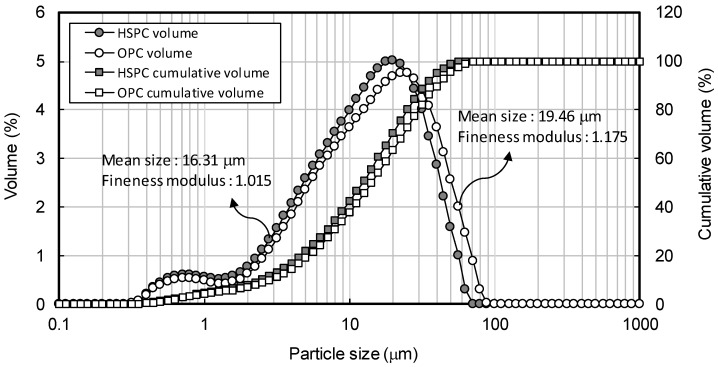
The particle size distribution of OPC (ordinary Portland cement) and HSPC (high SO_3_ Portland cement).

**Figure 3:**
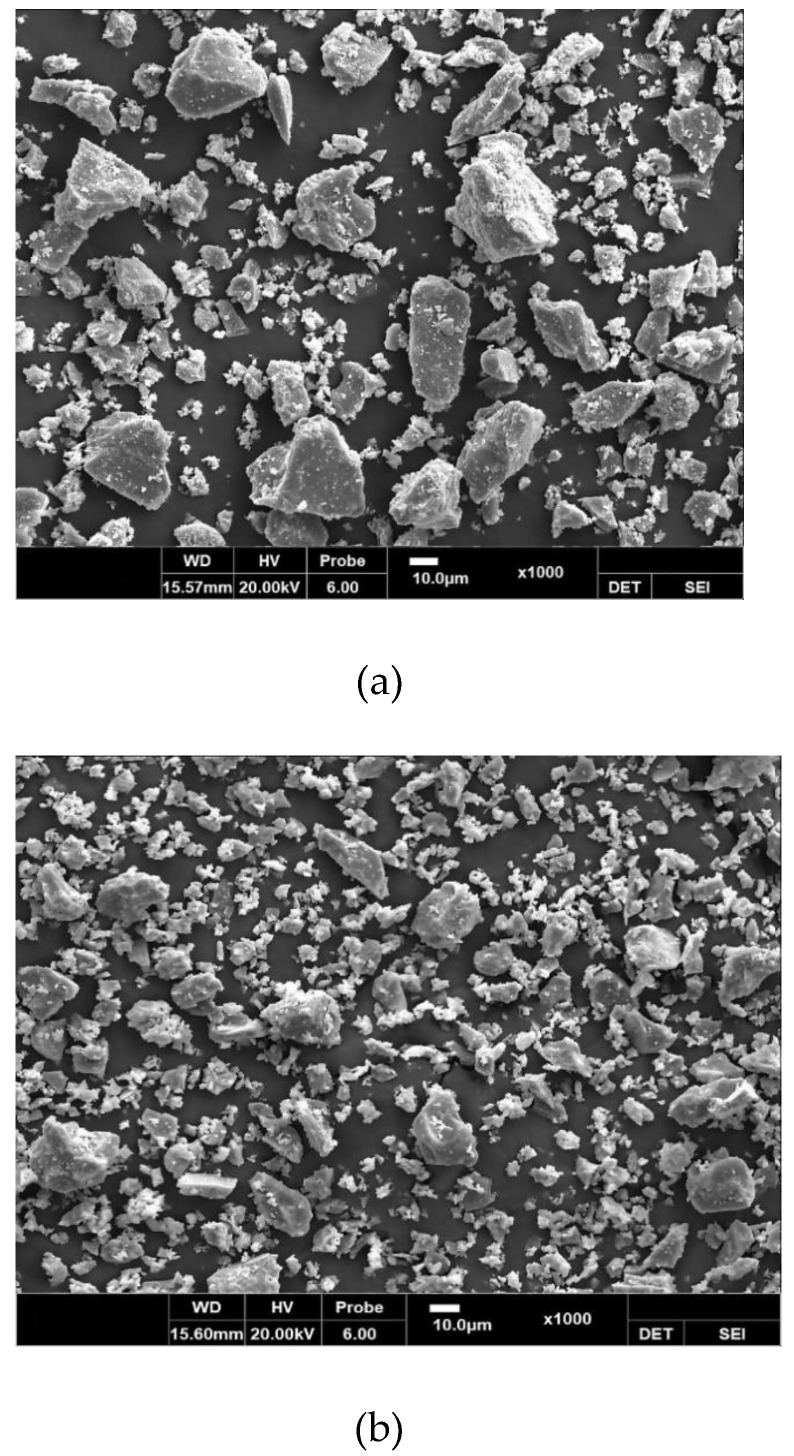
Scanning electron microscope micrograph: (**a**) OPC; (**b**) HSPC.

**Figure 4:**
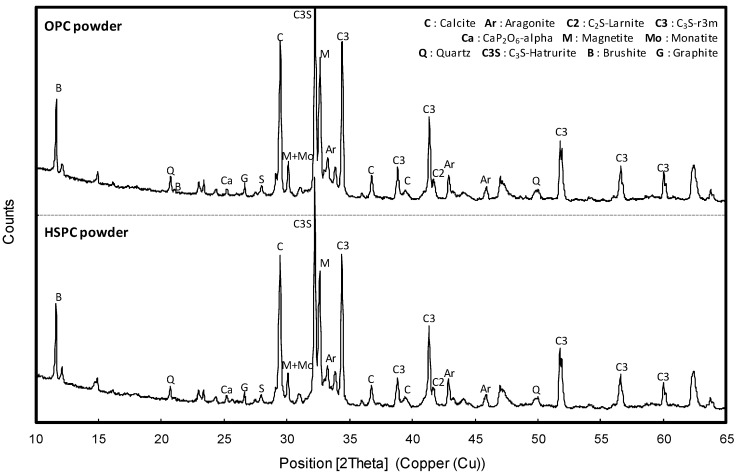
X-ray diffraction patterns of OPC and HSPC.

**Figure 5:**
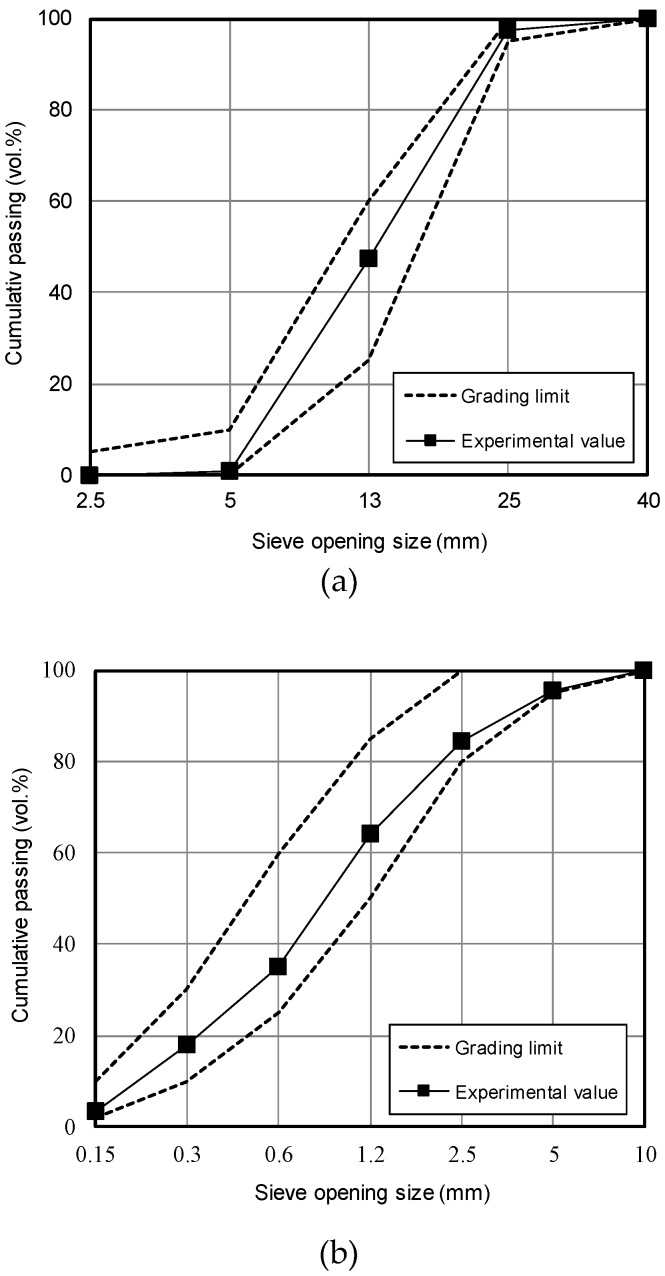
Particle distribution curves of aggregates used: (**a**) Coarse aggregates; (**b**) Fine aggregates.

Table 2 shows the chemical composition of binders used in the tests. HSPC was characterized by higher SO_3_ content (3.13%) and loss on ignition (L.O.I.) than OPC. The particle size distributions, obtained using a Mastersizer 2000 (Malvern Panalytical, Seongnam-si, Korea), are shown in Figure 2. The HSPC cement had a mean diameter of 16.31 μm and fineness modulus 1.015, while OPC had a diameter of 19.46 μm and fineness 1.175; i.e., HSPC had a smaller and finer particle size distribution than OPC. Analysis using a Genesis-2020 scanning electron microscope (SEM, Emcrafts, Gwangju-si, Korea) (Figure 3) confirmed this result, revealing similarly shaped particles, but smaller in the case of HSPC. Crystallinity was analyzed by X-ray diffraction (X’pert3 Powder PW 3050, Malvern Panalytical, Seongnam-si, Korea) (Figure 4); the samples had a very similar crystal structure, except that OPC contained more brushite. Figure 5 shows the particle size distribution curves of the aggregates used in this study.

### 2.2. Experimental Parameters

Two series of setting time and early compressive strength tests were carried out: Series I (mortar test); Series II (concrete tests). The tests, fineness level settings, and concrete curing conditions for the two types of cement are shown in Table 3. In Series II, cement curing was set to level 3 (chamber, indoor, sealed). All experiments were performed in an environment with air temperature 20 ± 2 °C and relative humidity 30%–40%, as recommended by the National Conference of Standards Laboratories–International (NCSLI). The Series I mortar mixing proportions are summarized in Table 4. Water/cement (W/C) and cement/sand (C/S) ratios (ASTM C778) [35] were set identically for the two types of cement, and the same cement, water, and superplasticizer (admixture, AD) masses were used to make the samples. The Series II concrete mixing proportions are shown in Table 5. The quantities in these were likewise set identically. The binder unit content was fixed at 330 kg/m^3^, which corresponded to the compressive strength most commonly used for a typical apartment floor (24 MPa). The ready-mixed concrete was produced by Remicon S companies (Busan, Korea), which delivered the concrete to the construction sites. Ten percent of the total binder quantity was replaced with fly ash to improve durability and long-term strength, by increasing cement mix fluidity, and a polycarboxylic superplasticizer-based admixture was used.

### 2.3. Experimental Procedure

**Figure 6:**
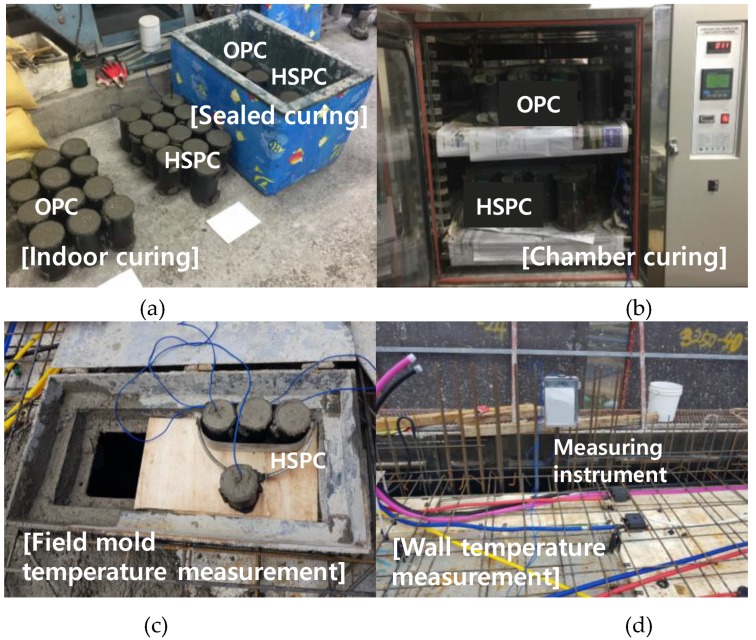
Photographs of the concrete curing test methods: (**a**) Indoor and sealed curing; (**b**) Chamber curing; (**c**) Mold temperature measurement; (**d**) Wall temperature measurement.

In Series I, the mortar flow test was performed according to ASTM C1437 [36], and the mortar setting test according to ASTM C403/C403M [37]. The compressive strength of mortar was measured at the age specified by ASTM C109/C109M [38]. In Series II, the concrete slump test was conducted in accordance with standard ASTM C143/C143M [39], and the air content test in accordance with ASTM C231/C231M [40]. Cylindrical specimens for compressive strength (Ø: 100 × 200 mm) were fabricated in accordance with the “Standard Test Method for Compressive Strength of Cylindrical Concrete Specimens”, described in ASTM C39/C39M [41], and the compressive strength by elapsed time was measured. As shown in Figure 6, T-type thermocouples were installed to measure the temperature history of the specimens under each curing condition using a data logger. The model proposed in ASTM C873/C873M [42] was installed to measure the compressive strength of concrete samples and record the actual history of hydration at an early age.

Equation (5), a functional formula proposed by Nurse–Saul, was used to calculate the sample maturity according to ASTM C1074 [43,44,45]. Maturity theory is closely related to the strength improvement of Portland cement concrete and was first proposed by Saul in 1951, who calculated the maturity index based on the lowest temperature at which the strength could be improved [46]. Maturity theory was further refined by Bergström [47].

(5) $$M\;=\;\sum (T\;-\;T_{0})\cdot \Delta t$$

where M is the maturity index (°C∙h), T is the average concrete temperature (°C) during the time interval Δt (h), T_0_ is the datum temperature (typically −10 °C), and t is the elapsed time (h).

Equation (6) was used to predict the early-age strength. It is a functional formula proposed by Plowman in 1956, based on the hypothesis that the compressive strength of concrete is close to linear when expressed as a logarithmic function of maturity using the Nurse–Saul equation [48]. As this simple equation has only two constants to be determined, it is generally applicable [49]:

f_c_ = a + b∙ln(M) (6)

where f_c_ is the compressive strength (MPa), M is the maturity index (°C∙h), and a and b are regression constants.

## 3. Results and Discussion

### 3.1. Setting Time and Early Strength Properties of Mortar

**Figure 7:**
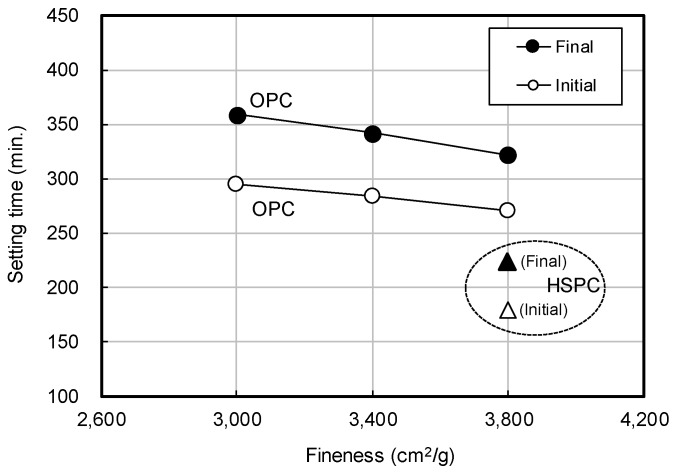
Setting time of OPC by fineness.

Figure 7 shows the OPC setting time by fineness. Initial setting time decreased by approximately 3 min, with fineness increased by 100 cm^2^/g, and the final setting time was reduced by 4 min 38 s. At the same fineness (3800 cm^2^/g), HSPC initial and final setting times were less than those of OPC by 92 and 98 min, respectively.

**Figure 8:**
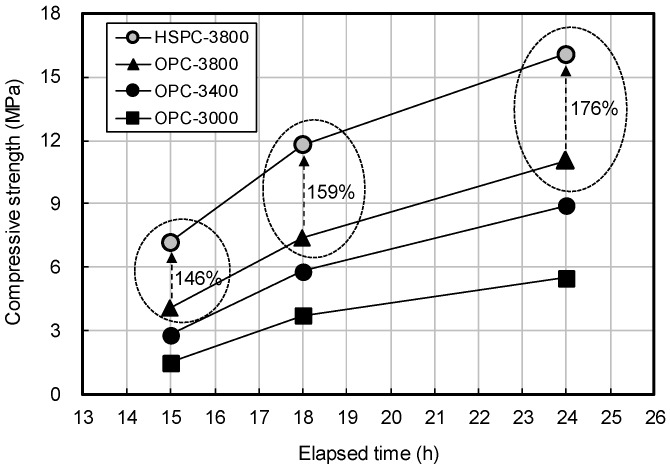
Early compressive strength of OPC and HSPC by elapsed time.

**Figure 9:**
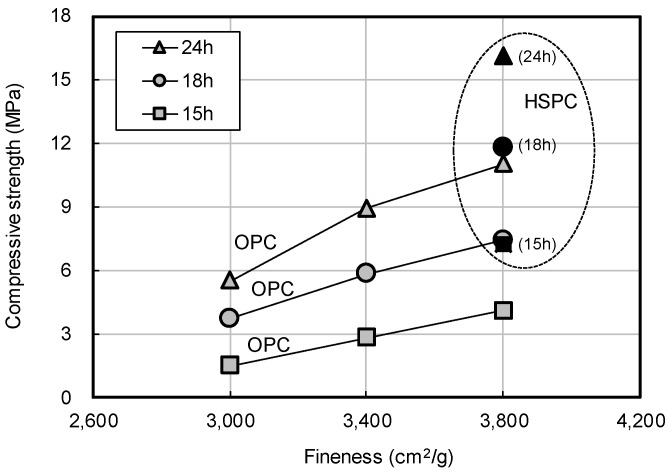
Early compressive strength of OPC by fineness.

Figure 8 shows the early compressive strength of OPC and HSPC by elapsed time. At the same fineness (3800 cm^2^/g), the compressive strength of HSPC was higher by 146%, 159%, and 176% after 15 h, 18 h, and 24 h, respectively. Figure 9 shows the early compressive strength of OPC by fineness. Early compressive strength increased with the degree of fineness. Strength improvements of 0.33 MPa, 0.46 MPa, and 0.69 MPa were observed at 15 h, 18 h, and 24 h of elapsed time, per 100 cm^2^/g increase in fineness.

**Figure 10:**
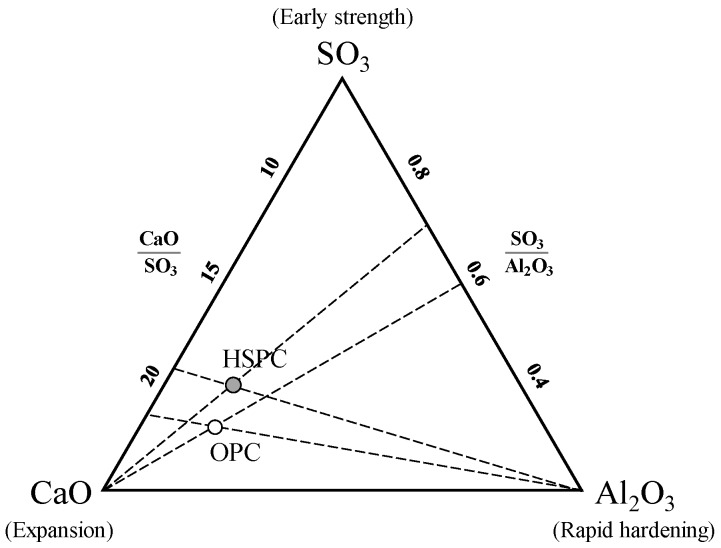
CaO/SO_3_ and SO_3_/Al_2_O_3_ molar ratios in OPC and HSPC.

**Figure 11:**
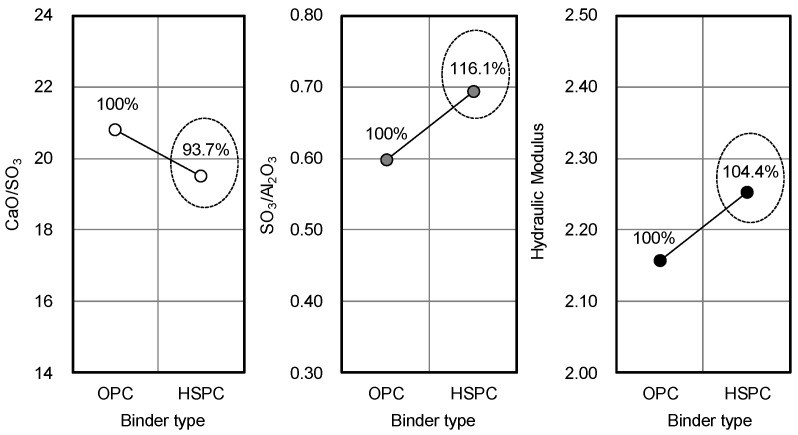
CaO/SO_3_, SO_3_/Al_2_O_3_, and hydraulic modulus molar ratios in OPC and HSPC.

Taken together, the results of Figure 7, Figure 8 and Figure 9 showed that as the fineness of cement increased, setting time decreased, and early compressive strength increased. This might be due to enhanced activation of the initial hydration reaction with increasing fineness. However, at the same level of fineness, HSPC exhibited a faster setting time and higher early compressive strength than OPC. For this reason, the molar ratios CaO/SO_3_ and SO_3_/Al_2_O_3_ and the hydraulic moduli of OPC and HSPC were reviewed (Figure 10 and Figure 11). For HSPC relative to OPC, the CaO/SO_3_ ratio was lower (93.7%), the SO_3_/Al_2_O_3_ ratio was higher (116.1%), and the hydraulic modulus (CaO/(SIO_2_ + Al_2_O_3_ + Fe_2_O_3_)) was also higher (104.4%). These differences have implied correspondence with the faster setting time and higher early compressive strength of HSPC at the same fineness.

### 3.2. Early Strength Properties of Concrete

Fresh OPC and HSPC were produced from the batchers at Remicon S. The initial air content and slump values after production were compared with those obtained one hour after production. The test results revealed that both OPC and HSPC met the target slump and air content values of 180 ± 25 mm and 4.5 ± 1.5%, respectively (Table 6).

**Figure 12:**
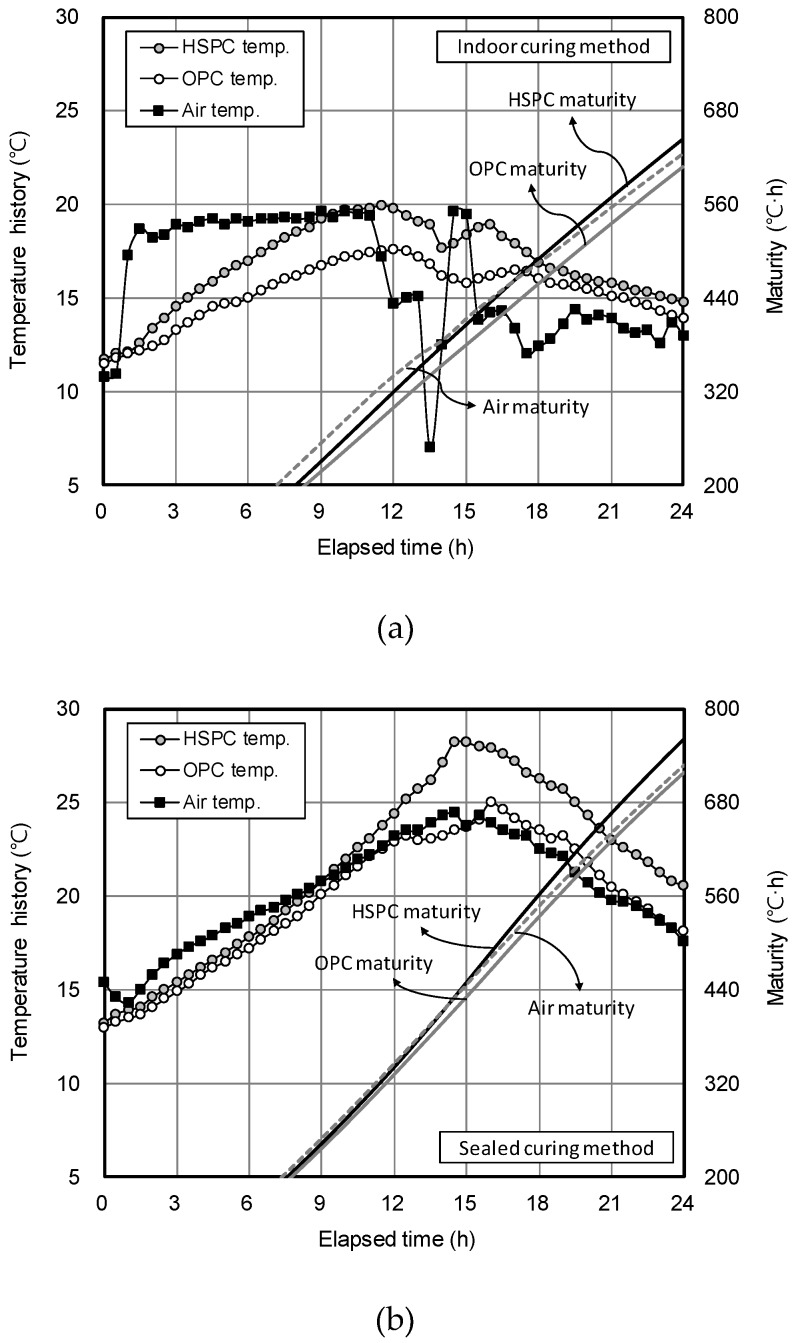

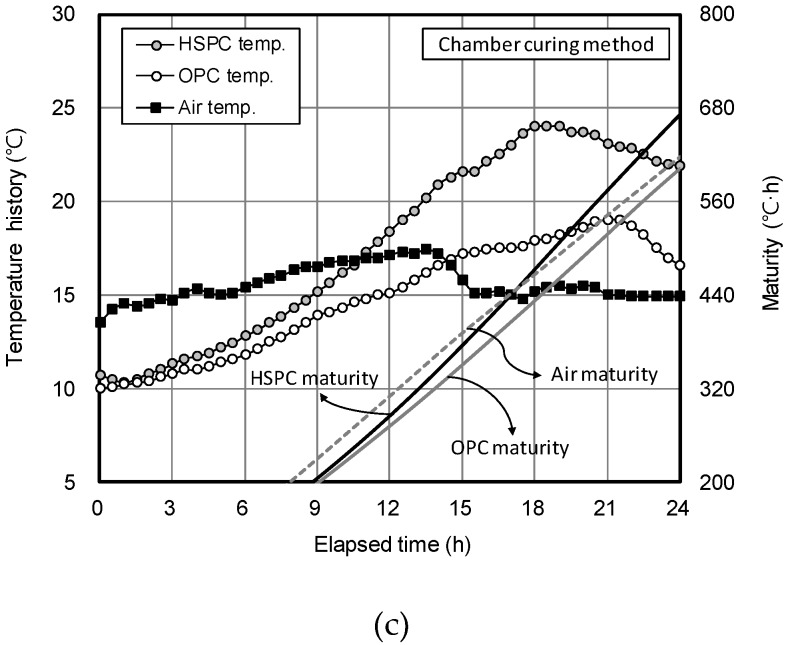
Concrete curing temperature history and maturity for different curing methods: (**a**) Indoor curing method; (**b**) Sealed curing method; (**c**) Chamber curing method.

Figure 12 shows the curing temperature history and maturity for concrete samples under the three curing methods. The order of maturity after 24 h was HSPC concrete > Air > OPC concrete, regardless of the method. The indoor curing results after 24 h (Figure 12a) showed that HSPC concrete maturity (644.6 °C·h) was 103% higher than air (625.8 °C·h), whereas OPC concrete (608.6 °C·h) was as low as 97.2%. The sealed curing results after 24 h (Figure 12b) showed that HSPC concrete maturity (762.5 °C·h) was 104.6% higher than air (729.2 °C·h), whereas OPC concrete (719.2 °C·h) was at 98.6%. The chamber curing results after 24 h (Figure 12c) showed that HSPC concrete maturity (672.7 °C·h) was 108.8% higher than air (618.6 °C·h), while OPC concrete (602.1 °C·h) was 97.3% of the air value.

**Figure 13:**
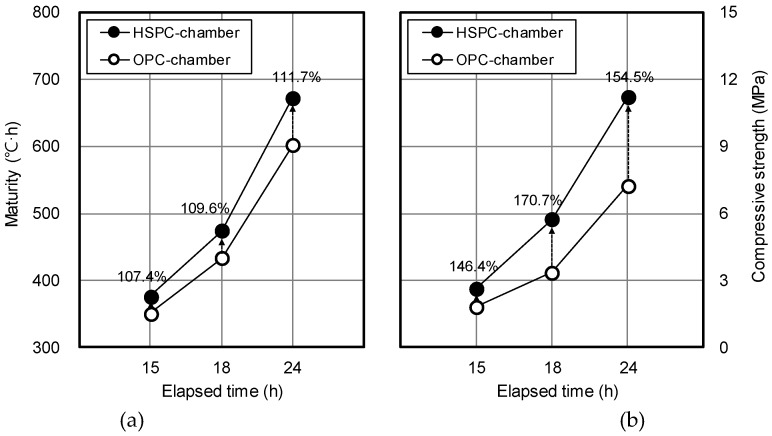
Comparison of maturity and early strength of OPC and HSPC concrete: (**a**) Maturity; (**b**) Early compressive strength.

**Figure 14:**
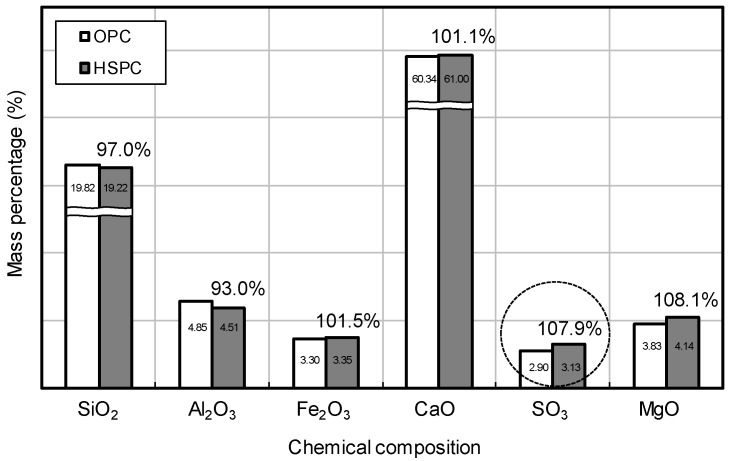
X-ray fluorescence analysis results of OPC and HSPC.

Figure 13 compares the maturity and early strength of OPC and HSPC concrete. At elapsed times of 15 h, 18 h, and 24 h, the maturity of HSPC concrete was 107.4%, 109.6%, and 111.7% higher than that of OPC concrete, and the early compressive strength was 146.4%, 170.7%, and 154.5% higher. The differences in HSPC over OPC were, firstly, higher fineness (111.8%), leading to early activation of the hydration reaction; secondly, higher SO_3_ content (107.9%) as measured by X-ray fluorescence analysis (Figure 14); thirdly, CaO/SO_3_ was low, and SO_3_/Al_2_O_3_ and hydraulic modulus were high (Figure 10 and Figure 11). As a result, the heat of hydration and maturity of HSPC concrete were higher than those of OPC under the same mixing and curing conditions (Figure 12), leading to higher early compressive strength.

**Figure 15:**
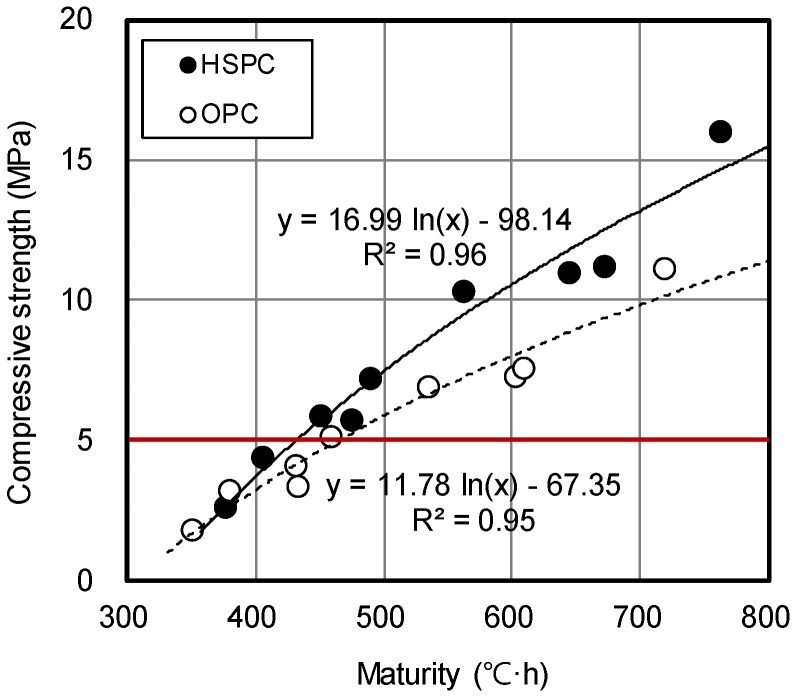
Derived strength enhancement curves of OPC and HSPC concrete.

Figure 15 shows the strength enhancement curves for OPC and HSPC concrete, derived by applying the functional formula proposed by Plowman (Equation (6)). The strength enhancement curves for OPC and HSPC are modeled as y = 11.78 ln(x) − 67.35 and y = 16.99 ln(x) − 98.14, respectively, with high correlations of 95% and 96%. At the maturity of 400–800 °C·h, the slope of the HSPC concrete strength enhancement curve was 144.2% that of OPC concrete. The most important element for reducing the number of cycles per floor for a typical apartment construction site is the time required to reach a compressive strength of 5 MPa, at which point form removal is possible [50]. OPC concrete reached 5 MPa at 465 °C·h, and HSPC concrete reached 5 MPa at 433 °C·h maturity; therefore, the application of HSPC to actual construction sites could enable form removal at approximately 6.9% lower maturity than OPC.

**Figure 16:**
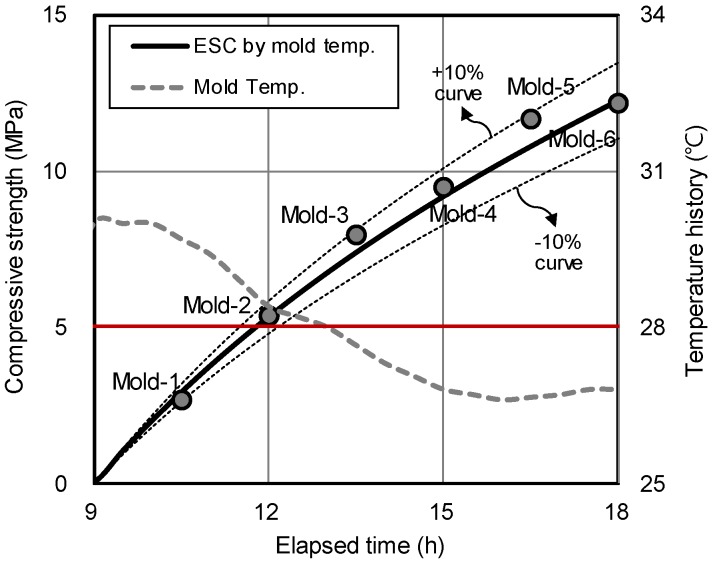
Comparison of the estimated strength curve (ESC) and measured compressive strength of the field mold of HSPC concrete.

Figure 16 compares the estimated strength curve (ESC) for HSPC concrete, based on the temperature history curve of the field molds, with the measured compressive strength of the field molds. The actual compressive strengths of all six field molds of HSPC were within ±10% of the ESC. Therefore, the reliability of the ESC for HSPC concrete was assumed to be 90% or higher.

**Figure 17:**
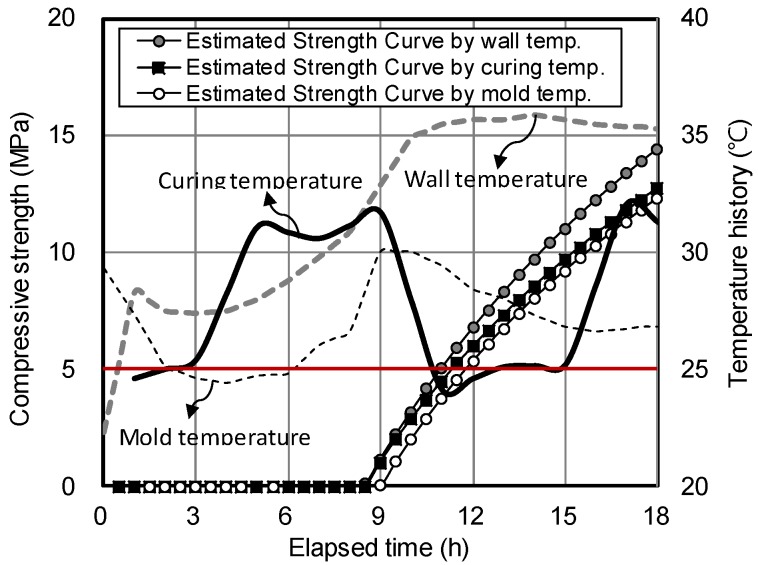
Estimated strength curve and temperature history for the structure wall, field mold, and curing air according to the curing elapsed time of HSPC concrete.

Figure 17 shows the ESC for the HSPC concrete samples according to the temperatures of the mold, curing air, and structure wall (200 mm thick). For a curing time of 9–18 h, the average maturity values of the field mold, curing air, and structure wall were 496 °C·h (96.6%), 514 °C·h (100%), and 550 °C·h (107%), indicating that curing in the structure wall resulted in approximately 10.8% higher hydration heat than the mold. Therefore, a safety factor of approximately 10.8% could be assumed if vertical form removal is performed at a time based on the attainment of 5 MPa compressive strength in the mold. The compressive strengths of the field mold, curing air, and structure wall reached 5 MPa after 11.8 h, 11.3 h, and 11 h, indicating that the applicable time of form removal for the structure wall was approximately 48 min earlier than that of the mold (i.e., without the 10.8% safety factor).

**Figure 18:**
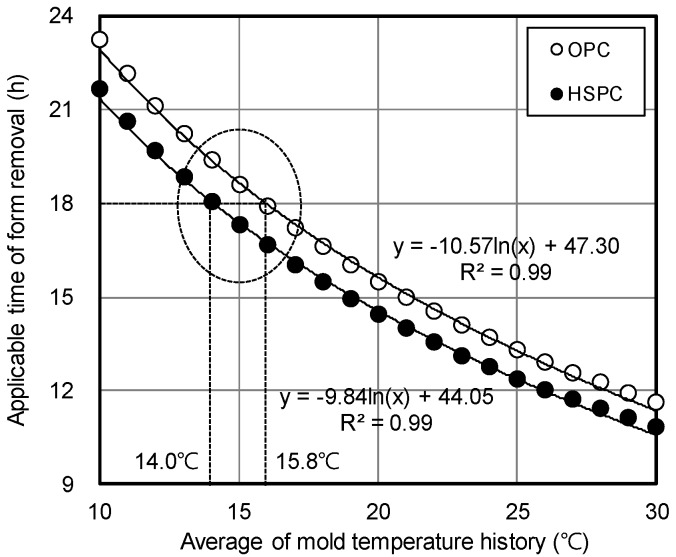
Applicable time of form removal according to the average temperature history of field mold.

Figure 18 shows the estimated applicable time of form removal according to the average temperature history of the concrete. Relationships of y = −10.57 ln(x) + 47.30 and y = −9.84 ln(x) + 44.05 were estimated for OPC and HSPC, respectively, with high correlations of 99.4% calculated for both. ACI 347R Guide to Formwork for Concrete [51] requires that the wall vertical formwork be retained for 12 h under conditions where the temperature of the air surrounding the concrete is above 50 °F (10 °C). However, in the case of domestic construction sites, the wall vertical formwork is often removed at 8 am the day after the ready-mixed concrete is poured, which is normally completed by 2 pm. Therefore, this study assumed that the target vertical form removal time was 18 h after concrete pouring. This required an average curing temperature greater than 15.8 °C for OPC and 14 °C for HSPC. Therefore, the strength required for form removal could be achieved at a 1.8 °C lower curing temperature by employing HSPC concrete. As the curing temperature might be reduced during construction in winter, this would enable a reduction of heat curing costs.

**Figure 19:**
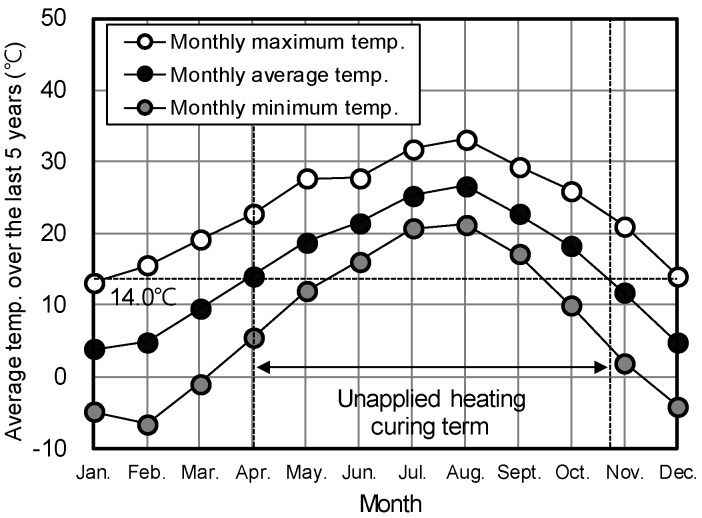
Months when heat curing would not be required in Busan due to using HSPC concrete.

Figure 19 illustrates the length of time for which heat curing would not be required to ensure the strength needed for form removal (5 MPa) within 18 h after concrete pouring by applying HSPC. This length of time was calculated by analyzing the monthly average temperatures of Busan in Korea for the last five years. Busan is a metropolitan city in the south of Korea and has a project that will actually apply HSPC. It was found that heat curing would not be necessary from early April to the end of October (approximately 6.8 months) when using HSPC; however, measures, such as heat curing, would be required for the remaining 5.2 months to ensure an average curing temperature of 14 °C or higher.

## 4. Conclusions

In this study, the influences of chemical composition and fineness on the development of concrete strength by curing conditions were investigated through the performance evaluation of HSPC and OPC. The early compressive strength of HSPC was predicted, and its application to actual construction sites was examined. The main conclusions are as follows.

(1) According to the Series I (mortar) test, increasing the fineness of OPC resulted in faster setting time and early compressive strength. At the same fineness (3800 cm^2^/g), the initial and final setting times of HSPC were 92 and 98 min less than OPC, and the early compressive strength was approximately 176% higher after 24 h curing.
(2) HSPC CaO/SO_3_ molar ratio was lower (93.7%), SO_3_/Al_2_O_3_ was higher (116.1%), and the hydraulic modulus was 104.4% higher than OPC.
(3) According to the Series II (concrete) test, the maturity at elapsed time 24 h was HSPC > Air > OPC, regardless of the curing method. At elapsed times of 15 h, 18 h, and 24 h, the maturity of HSPC concrete was 107.4%, 109.6%, and 111.7% higher than OPC concrete, and the early compressive strengths were 146.4%, 170.7%, and 154.5% higher.
(4) The reasons for the improvement were that the fineness of HSPC was approximately 111.8% (400 cm^2^/g increase) greater than OPC, leading to early activation of the hydration reaction, and by the results of X-ray fluorescence analysis, HSPC had 107.9% higher SO_3_ content. The heat of hydration and maturity of HSPC were higher than that of OPC under the same mixing and curing conditions.
(5) The applicable time of form removal was estimated according to the average temperature history of the field molds. Relationships of y = −10.57 ln(x) + 47.30 and y = −9.84 ln(x) + 44.05 were estimated for OPC and HSPC concrete, respectively. The average curing temperature must be 15.8 °C or higher for OPC and 14 °C or higher for HSPC to ensure 5 MPa within 18 h after concrete pouring.

Based on the monthly average temperatures in Busan, Korea, for the last five years, heat curing would not be required from early April to the end of October (approximately 6.8 months). During the remaining months, measures, such as heat curing, would be required to maintain average curing temperatures of 14 °C or higher for HSPC.

In future studies, the performance impact of HSPC according to mineralogical composition would be investigated, and the effects of the construction period and cost reduction effects would be examined when HSPC is applied to actual construction sites.

## Figures and Tables

**Table 1:** Physical properties of the materials used in the tests. (OPC, ordinary Portland cement; HSPC, high SO_3_ Portland cement.).

Materials	Physical Properties
OPC	Ordinary Portland cement (density: 3.15 g/cm^3^, fineness: 3400 cm^2^/g)
HSPC	High SO_3_ Portland cement (density: 3.13 g/cm^3^, fineness: 3800 cm^2^/g)
FA	Fly ash (density: 2.20 g/cm^3^, fineness: 3850 cm^2^/g)
Fine aggregate	Crushed sand 60% (density: 2.63 g/cm^3^, absorption: 1.30%)
	Washed sea sand 40% (density: 2.60 g/cm^3^, absorption: 1.34%)
Coarse aggregate	Crushed granitic aggregate (size: 25 mm, density: 2.65 g/cm^3^, absorption: 0.89%)
Admixture	Polycarboxylic superplasticizer-based type (density: 1.26 g/cm^3^)

**Table 2:** Chemical composition of binders used in the tests. (OPC, ordinary Portland cement; HSPC, high SO_3_ Portland cement.).

Materials	Chemical Composition (%)								L.O.I. ^(1)^
	SiO_2_	Al_2_O_3_	Fe_2_O_3_	CaO	MgO	SO_3_	K_2_O	Other	
OPC	19.82	4.85	3.30	60.34	3.83	2.90	1.08	0.86	3.02
HSPC	19.22	4.51	3.35	61.00	4.14	3.13	1.04	0.79	2.82

^(1)^ L.O.I.: Loss on ignition.

**Table 3:** Experimental parameters for setting time and early compressive strength tests using ordinary Portland cement (OPC) and high SO_3_ Portland cement (HSPC).

Series	Type	Experimental Factors	Experimental Levels	Measured Parameters
I	Mortar	Cement fineness and type	3000 cm^2^/g (OPC)	Setting time (h) Compressive strength (MPa)
			3400 cm^2^/g (OPC)	
			3800 cm^2^/g (OPC, HSPC)	
II	Concrete	Cement fineness and type	3400 cm^2^/g (OPC)	Slump (mm) Air content (%) Compressive strength (MPa) Maturity (°C·h)
			3800 cm^2^/g (HSPC)	
		Curing type	Chamber (15 °C)	
			Indoor (variable temperature)	
			Sealed (variable temperature)	

**Table 4:** Mixing proportions for mortar tests.

Series		W/C (%)	C:S ^(1)^	Cement (g)	Water (g)	AD ^(2)^ (B×%)
**I** **(Mortar)**	OPC	50	1:3	450	225	0.7
	HSPC	50	1:3	450	225	0.7

^(1)^ C:S = Cement:sand (ISO standard sand); ^(2)^ AD: Admixture. W/C: water/cement.

**Table 5:** Mixing proportions for concrete tests.

Series		W/B ^(1)^ (%)	S/a ^(2)^ (%)	Unit Weight(kg/m^3^)								AD ^(8)^ (B×%)
				B ^(3)^	W ^(4)^	OPC	HSPC	FA	S1 ^(5)^	S2 ^(6)^	G ^(7)^	
**II** **(Concrete)**	**OPC**	**50**	**49.2**	330	165	300	0	30	541	361	932	0.8
	HSPC	50	49.2	330	165	0	300	30	541	361	932	0.8

^(1)^ W/B: Water/Binder; ^(2)^ S/a: Sand/aggregates; ^(3)^ B: Binder; ^(4)^ W: Water; ^(5)^ S1: Crushed sand; ^(6)^ S2: Sea sand; ^(7)^ G: Gravel; ^(8)^ AD: Polycarboxylic superplasticizer-based type admixture.

**Table 6:** Properties of fresh concrete.

Series		Air (%)		Slump (mm)	
		Initial	1 h	Initial	1 h
**II** **(Concrete)**	OPC	4.6	4.3	165	170
	HSPC	3.8	3.5	185	185
